# Defensive pharmacy practice: a gap in our understanding

**DOI:** 10.1007/s11096-021-01285-4

**Published:** 2021-06-13

**Authors:** Juliette O’Connell

**Affiliations:** School of Pharmacy and Pharmaceutical Sciences, Trinity College Dublin, Dublin, Ireland

**Keywords:** Defensive medicine, Defensive practice, Pharmacy practice, Social pharmacy

## Abstract

Defensive practice is prevalent across healthcare disciplines and much study has been performed on this behaviour in medicine and nursing. However, little research has been carried out on defensive practice in pharmacy, despite its potential to increase healthcare costs, reduce quality of care and affect pharmacist job satisfaction. With a more litigious society emerging and greater level of regulation, the pharmacy profession shares many of the influences of defensive practice identified in other healthcare professions. As a result, pharmacists too may engage in defensive practice behaviours in order to protect themselves from the perceived risk of litigation. Research in this area is necessary to identify how this phenomenon affects the profession and to develop methods of improving pharmacy practice. While this type of research would not be without challenges, it could form the basis for policy change and greater professional representation, ultimately improving quality of care for patients.

## Introduction

Defensive practice, or defensive medicine, has a number of definitions across the literature, primarily influenced by the healthcare profession under study. Perhaps the broadest definition of defensive practice is “deviation from sound medical practice that is induced primarily by a threat of liability” [1]. Defensive practice involves both assurance (positive) behaviours, e.g. ordering additional tests, referral to another healthcare practitioner or prescription of additional medications, and avoidance (negative) behaviours e.g. avoiding a particular field of work or avoiding certain patients identified as high-risk. Defensive practice has a number of negative consequences; it can lead to increased healthcare spending, reduced quality of care to patients and reduced job satisfaction among practitioners [2, 3].

In the existing literature, defensive medicine has been identified across disciplines, with primary focus on the practice of physicians and nurses and particular emphasis on the area of obstetrics and gynaecology. However, defensive practice is a concept which can affect all allied health professionals, especially with the emergence of a more litigious society and the marketisation of healthcare [4]. To date, little research has been carried out examining defensive practice among pharmacists. Broom et al. [5] included pharmacists in a focus group examining defensive use of antibiotics, but data specific to the role of pharmacy in this study was limited. As a result, defensive pharmacy practice is an under-investigated area of research which has potential to impact negatively on patients, pharmacists and the healthcare service at large.

### Pharmacy regulation and defensive practice

In Ireland, prior to the introduction of the revised Pharmacy Act 2007, pharmacists were not subject to statutory rules regulating disciplinary procedures and fitness to practice [6]. Change in professional regulation was both desirable and necessary for the benefit of patients and protection of the public. However, this change introduced the possibility for a pharmacist to fear litigation and disciplinary proceedings, potentially leading to defensive practice to safeguard their registration and protect their livelihood, where previously it had been difficult if not impossible to remove a pharmacist from the register [7]. In Idaho, it has been identified that pharmacy is governed by prescriptive rule-based regulation rather than regulation based on standards of care [8]. The lack of flexibility enforced by prescriptive regulation could potentially encourage defensive practice. In the United Kingdom, regulatory constraints were identified to create situations where pharmacists felt unable to act according to their professional values [9]. Regulation of the pharmacy profession could encourage defensive practice among pharmacists.

### Defensive pharmacy practice

Little research has investigated the drivers and behaviours of defensive pharmacy practice. However, it may be hypothesised that drivers are similar to those identified in the literature among other health professionals. Influences identified in the literature include risk of litigation, peer pressure, education by senior colleagues, practice norms, pressure by patients and pressure from regulatory bodies [3, 5, 10–12]. It is possible that the drivers of defensive practice are context- and setting-specific in pharmacy, particularly as it is a profession which encompasses several different areas of practice. For example, the drivers and behaviours of defensive practice in a community pharmacy setting could be different to that in a hospital pharmacy setting.

One influence which has particular weight in community pharmacy practice is the marketisation of healthcare. This has been identified as a powerful driver of defensive practice in other areas [4]. Consumerism and a move away from paternalistic medicine has created a climate where patients demand excellent “customer service” from their healthcare providers and have higher expectations of care and reduced tolerance for errors [4]. This is not wholly negative—patients should be in a position to ask for and receive high quality care. However, the model of community pharmacy lends itself to greater pressure as it also performs as a retail unit, and it can be difficult for patients to distinguish between the retail side and the healthcare provision side and may expect pharmacy healthcare provision to follow the specifications of a rational market. However, as identified by Hoffman [13], healthcare cannot function as a rational market, as purchasing healthcare is not the same as purchasing other consumer goods. In the hospital pharmacy setting, there may be a different kind of influence—that of the chain of command and “medical manners” [5]. Pharmacists have identified that while they can advise on best practice, they are often not in a position to enforce this. Thus, the value of collegiality could influence defensive behaviours [5]. In addition, practitioners may avoid getting involved in the care of a patient under another clinician’s care for fear they may be implicated in litigation arising from this care [10].

Managing the uncertainty of clinical practice can also have an influence on defensive practice behaviours. Both Cunningham and Wilson [14] and Vento et al. [2] identify the current model of biomedicine is underpinned by assumptions of polarised, clear-cut “correct” and “incorrect” diagnoses, treatment options and outcomes, when in fact, medicine is an uncertain art subject to the influence of practitioner and patient ideas, beliefs and context. The polarised assumption of this model implies that, given sufficient knowledge, a healthcare professional will always make the “correct” decision [14]. This assumption can be damaging, both to the professional as they internalise the feeling of failure, and to their patients, who may then receive care which is defensive.

The influences on defensive practice then lead to behaviours. The types of behaviours employed in defensive pharmacy practice have not previously been identified. However, they may be hypothesised based on behaviours observed among other healthcare professionals. Negative defensive pharmacy practice may include increased referral to other healthcare practitioners, overly conservative safety netting strategies, or avoidance of treatment of certain conditions or supplying certain services, especially to high-risk patients. Positive defensive pharmacy practice may involve performing or recommending tests which are not medically indicated, increased follow up, increased consultation of specialists, engagement in more detailed note taking, provision of more detailed explanations of procedures to patients or developments of additional audits within practice.

### Cost of defensive practice

While difficult to truly assess the cost of defensive medicine, it has been estimated to be €10–12 billion per year in Italy [2, 15]. The medical liability system in the United States costs $55.6 billion per year [2]. While estimates of the costs associated with defensive medicine and defensive practice are not available more generally, it is reasonable to assume that the practice contributes to healthcare spending worldwide, since the cornerstone features of defensive practice have inherently high costs associated with them. This is certainly an area where defensive pharmacy practice can contribute to increased healthcare spending, but also offers potential for an area where greater education of pharmacists and pharmacy staff can help reduce the financial burden on the healthcare system, by encouraging judicious use of strategies like referrals.

Further empowerment of pharmacists in exercising their clinical judgement could help to reduce defensive practice as their roles expand beyond the traditional processes of dispensing and counselling. As pharmacists become more involved in vaccination provision and screening services, the potential for greatly enhancing patient care is evident. However, pharmacists must feel supported to achieve this outcome and to reduce defensive practice.

### Potential for practice improvement

Developing an understanding of defensive practice in pharmacy has the potential to inform initiatives addressing the practice. Completing extensive research into defensive practice in the pharmacy profession could inform future policy and legislation which both protects patients and empowers pharmacists to provide high quality care, without fear of litigation motivating unnecessarily conservative approaches to practice.

Establishment of professional bodies, not as regulators but as an advocate for the professionals themselves could help empower pharmacists in exercising their clinical judgement and reduce the fear that could spawn defensive practice.

Education initiatives could improve pharmacy practice in this area. It is possible that pharmacists are unaware of the influences on their practice that lead to defensive behaviours. It has been identified that education is a missed opportunity to reduce defensive practice [10].

It has also been identified that uncertainty around complaints procedures can cause additional stress on practitioners, so making the complaints process clearer, more transparent and less punitive could help healthcare professionals who are engaging in defensive practice [16].

Following expansion of knowledge about defensive pharmacy practice, a potential way forward could include moving away from prescriptive regulation of pharmacy and towards a standard of care model of regulation, as suggested by Adams [8]. Revising the regulation model governing pharmacy could offer pharmacists the flexibility needed to address the individuality of scenarios encountered in practice without fear of legislative implications.

### Challenges

Numerous challenges to research in the area of defensive pharmacy practice may be significant. Under-reporting of defensive practices may occur due to social desirability bias [17]. Pharmacists may identify some of their own practices as defensive but may not report these behaviours when asked as they do not want to appear a suboptimal practitioner. This can be limited by ensuring anonymity of professionals during a web-based survey, though this would be more difficult in focus groups or semi-structured interviews. Defensive practice has been described as an “open secret”—widely known but rarely acknowledged [18]. Such may be the case in pharmacy practice also.

Validating an instrument to measure defensive practice among pharmacists could be difficult; so much of defensive practice depends on an individual’s perspective, perception of risk and personal motivations. Variance in definitions around defensive practice (Table 1) also makes it difficult to pinpoint a “gold standard” measure of defensive practice. One potential solution would be to employ a nominal group technique consensus process across a range of pharmacy professionals, though this may be limited to identifying ideal practice across a limited range of clinical vignettes and would not allow for the wide variety of scenarios encountered by pharmacists in day-to-day practice. In addition, as identified by Whittaker and Havard [18] in a study of defensive practice in social work, using active practitioners to rate defensive behaviours can be biased by their own experiences. Like the social work students in this study, pharmacists that view themselves as ethically sound practitioners may feel uncomfortable in rating a behaviour they engage in as defensive, as it may reveal they participate in undesirable practices [18]. Whittaker and Havard [18] highlight a key challenge in the area of defensive practice research—that practitioners often do not agree on what qualifies as defensive practice, and while it is often defined as a deliberate behaviour, it could be a less conscious process.

**Table 1:** Definitions of defensive medicine/practice in the literature

Definition	Discipline	Author(s)
Deviation from sound medical practice that is induced primarily by a threat of liability	Medicine	Studdert et al. [1]
Practices which are deliberately chosen in order to protect the professional worker, at the possible expense of the well-being of the client	Social work	Whittaker and Havard [18]
Deviation from sound medical practice that physicians engage in primarily because they perceive a threat of liability	Medicine	O’Leary et al. [10]
Departing from normal medical practice as a safeguard from litigation. It occurs when a medical practitioner performs treatment or procedure to avoid exposure to malpractice litigation	Medicine	Sekhar and Vyas [19]
Deviation from routine medical care in order to avoid or reduce the risk of real or perceived future legal consequences	Medicine	Borgan et al. [3]

In addition, much of the existing research in this area has been conducted in doctors and using the same instruments for pharmacists may fail to identify profession-specific influences and behaviours. Qualitative research would be helpful to gather this information and optimise research in this group.

## Conclusion

Defensive practice among pharmacists is under-investigated but could be impacting on quality of patient care and negatively impacting job satisfaction. Further research is required among pharmacists in order to identify the influences and behaviours of the phenomenon in this profession. It is highly unlikely that pharmacists are immune to this practice, evidenced by its pervasiveness across multiple other professions, including medicine, nursing, midwifery and social work. Like defensive practice in other professions, defensive pharmacy practice is possibly an “open secret”. Understanding the knowledge, influences and behaviours of pharmacists towards defensive practice will help to inform future policy and education initiatives. By beginning a conversation about it, defensive practice may be identified and addressed, improving both the quality of care for patients and the job satisfaction of pharmacists.

## Data Availability

Data sharing not applicable to this article as no datasets were generated or analysed during the current study. Not available.

## References

[1] Studdert DM, Mello MM, Sage WM, DesRoches CM, Peugh J, Zapert K (2005). Defensive medicine among high-risk specialist physicians in a volatile malpractice environment. JAMA.

[2] Vento S, Cainelli F, Vallone A (2018). Defensive medicine: it is time to finally slow down an epidemic. World J Clin Cases.

[3] Borgan SM, Romeus L, Rahman S, Asmar A (2020). Internal
medicine residents and the practice of defensive medicine: a pilot study across
three Internal Medicine Residency Programs. Cureus.

[4] Jani A, Papanikitas A, Feiler T, Hordern J, Papanikitas A (2018). “More than my job is worth”—defensive medicine and the marketisation of healthcare. Marketisation,
ethics and healthcare: policy, practice and moral formation.

[5] Broom A, Kirby E, Gibson AF, Post JJ, Broom J (2017). Myth, manners, and medical ritual: defensive medicine and the fetish of antibiotics. Qual Health Res.

[6] Purcell D (2004). Competition and regulation in the retail pharmacy market. Studies in public policy.

[7] Wingfield J, Weedle P (2008). From leadership and development to regulatory body—the Irish solution. Pharm J.

[8] Adams AJ (2019). Transitioning pharmacy to “standard of care” regulation: Analyzing how pharmacy regulates relative to medicine and nursing. Res Soc Adm Pharmacy.

[9] Astbury JL, Gallagher CT (2020). Moral distress among community pharmacists: causes and achievable remedies. Res Soc Adm Pharmacy.

[10] O'Leary KJ, Choi J, Watson K, Williams MV (2012). Medical students' and residents' clinical and educational experiences with defensive medicine. Acad Med.

[11] Annandale E (1996). Working on the front-line: risk culture and nursing in the New NHS. Sociol Rev.

[12] Wier J (2017). Protecting the public: an investigation of midwives perceptions of regulation and the regulator. Midwifery.

[13] Hoffman DR. The effect of patient demands and defensive medicine on health care costs [Internet]; 2015 [cited 20 Oct 2020]. https://www.inquirer.com/philly/blogs/healthcare/The-effect-of-patient-demands-and-defensive-medicine-on-health-care-costs.html.

[14] Cunningham W, Wilson H (2011). Republished original viewpoint: complaints, shame and defensive medicine. Postgrad Med J.

[15] Gelmetti C (2016). Cruising between Scylla and Charybdis … Just a hope?. Eur J Intern Med.

[16] Bourne T, Vanderhaegen J, Vranken R, Wynants L, De Cock B, Peters M (2016). Doctors' experiences and their perception of the most stressful aspects of complaints processes in the UK: an analysis of qualitative survey data. BMJ Open.

[17] Althubaiti A (2016). Information bias in health research: definition, pitfalls, and adjustment methods. J Multidiscip Healthc.

[18] Whittaker A, Havard T (2016). Defensive practice as 'fear-based' practice: social work's open secret?. Br J Soc Work.

[19] Sekhar MS, Vyas N (2013). Defensive medicine: a bane to healthcare. Ann Med Health Sci Res.

